# Antimicrobial Drug Resistance in Singapore Hospitals

**DOI:** 10.3201/eid1312.070299

**Published:** 2007-12

**Authors:** Li-Yang Hsu, Thean-Yen Tan, Roland Jureen, Tse-Hsien Koh, Prabha Krishnan, Raymond Tzer-Pin Lin, Nancy Wen-Sin Tee, Paul Ananth Tambyah

**Affiliations:** * National University of Singapore, Singapore; † Changi General Hospital, Singapore; ‡ Alexandra Hospital, Singapore; § Singapore General Hospital, Singapore; ¶ Tan Tock Seng Hospital, Singapore; # KK Women’s & Children’s Hospital, Singapore; 1 These authors contributed equally to this article.

**Keywords:** microbial drug resistance, surveillance, Singapore, nosocomial infections, dispatch

## Abstract

A new national antimicrobial resistance surveillance program in Singapore public hospitals that uses WHONET detected high levels of methicillin resistance among *Staphylococcus aureus* (35.3%), carbapenem resistance among *Acinetobacter* spp. (49.6%), and third-generation cephalosporin resistance among *Klebsiella pneumoniae* (35.9%) hospital isolates in 2006. Antimicrobial drug resistance is a major problem in Singapore.

Bacterial antimicrobial drug resistance is a worldwide problem that is exacerbated by the diminishing number of new antimicrobial drugs in the pharmaceutical pipeline (*1*,*2*). This is an emerging public health problem, especially in hospitals of the newly industrialized countries of Asia and the Pacific. In 2001, the World Health Organization (WHO) launched the first global strategy to counter this phenomenon (*3*), a key component of which is the development of surveillance programs to monitor trends in antimicrobial drug resistance and use (*3*).

Overarching surveillance programs monitoring antimicrobial drug–resistance trends on a national or regional level are present in Australia (*4*) and Europe (*5*). Such is not the case in Singapore, where surveillance efforts have generally been conducted only at the institutional level, with limited sharing and analysis of data. As a result, the actual scale of local antimicrobial drug resistance is not well defined. The Network for Antimicrobial Resistance Surveillance (Singapore), a voluntary group of healthcare professionals, was established in December 2005 to fill this gap.

## The Study

A laboratory-based surveillance program was established in 2006 to monitor the antimicrobial drug–resistance trends of 6 common nosocomial pathogens: *Staphylococcus aureus*, *Escherichia coli*, *Enterococcus* spp., *Klebsiella pneumoniae*, *Pseudomonas aeruginosa*, and *Acinetobacter* spp. Excluding coagulase-negative staphylococci, these organisms collectively account for >90% of positive bacterial cultures from nosocomial infections locally.

All 6 public sector acute-care hospitals in Singapore—2 tertiary-care hospitals, 3 secondary-care hospitals, and 1 institution dedicated to pediatrics and obstetrics/gynecologic services only—participated in the program. These hospitals constitute ≈76.5% of the 8,205 acute-care hospital beds available in the country (*6*).

All clinical isolates submitted to the externally accredited microbiology laboratories of these hospitals in calendar year 2006 were recorded for this study. Four laboratories performed antimicrobial drug–susceptibility testing predominantly through disk-susceptibility testing, supplemented by VITEK 2 system (bioMérieux, Marcy l’Etoile, France), following guidelines of the Clinical Laboratory Standards Institute (CSLI) (*7*). One laboratory used the VITEK 2 system exclusively, following CLSI guidelines (*7*), and the sixth laboratory used disk-susceptibility testing, following guidelines for the calibrated dichotomous sensitivity method (*8*).

Microbiologic and demographic data were extracted every quarter from the laboratory information system of each participating institution and converted into a standard format by using WHONET 5 (WHO, Geneva, Switzerland). Data were collated and analyzed centrally, with duplicates eliminated according to CLSI guidelines (*9*). Hospital bed occupancy data were obtained from the published records of each institution.

Statistical analysis was performed by using Excel 2003 (Microsoft, Redmond, WA, USA). Clinical microbiologists of the respective hospitals verified the analyzed data. Combined antimicrobial drug–susceptibility data were analyzed for the target organisms in 3 ways: for all isolates, for blood culture isolates only, and for isolates from intensive care unit (ICU) settings. The same analysis was also separately performed for data from each institution.

The distribution of resistant organisms isolated in 2006 is shown in the Table. The incidence density of resistant organisms from clinical samples for 2006 is shown in the Figure. Antimicrobial drug resistance was generally more prevalent in ICUs, but there was marked interhospital variation in resistance percentages. The tertiary hospitals had high rates of antimicrobial drug resistance, whereas the pediatric and women’s hospital had much lower rates.

**Figure:**
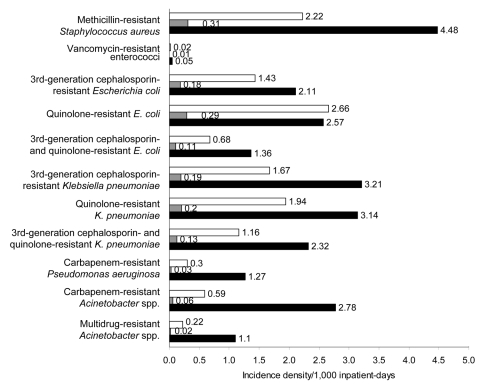
Incidence density of various antimicrobial drug–resistant bacteria isolated in public sector hospitals, Singapore, 2006. White bars, incidence density, all isolates (per 1,000 inpatient-days); gray bars, incidence density, blood isolates (per 1,000 inpatient-days); black bars, incidence density, intensive-care unit (ICU) isolates (per 1,000 ICU inpatient-days). *S. aureus, Staphylococcus aureus; E. coli, Escherichia coli; P. aeruginosa, Pseudomonas aeruginosa*.

Antimicrobial drug resistance in the Enterobacteriaceae was prevalent for amoxicillin-clavulanate (*K. pneumoniae* 36.0%, *E. coli* 26.7%), ciprofloxacin, and third-generation cephalosporins (Table). Imipenem resistance was present in 0.2% (14 isolates) of *K. pneumoniae.* Ertapenem resistance was reported in 0.2% of all *E. coli* isolates and 0.9% of all *K. pneumoniae* isolates at the institutions that routinely test for this agent.

**Table:** Drug-resistant clinical bacterial isolates cultured at public sector hospitals, Singapore, 2006*

Isolates	All resistant isolates			Resistant blood isolates				Resistant ICU isolates		
	No. (%) of all isolates†	% Range for single hospitals‡		No. (%) of all blood isolates†	% Range for single hospitals‡	p value§		No. (%) of all ICU isolates†	% Range for single hospitals†	p value¶
Methicillin-resistant *S. aureus*	3,517 (35.3)	18.0–44.3		497 (39.8)	23.8–44.4	<0.01		261 (46.7)	26.8–70.5	<0.01
Vancomycin-resistant enterococci (*E. faecium* or *E. faecalis*)	31 (0.8)	0–1.3		5 (1.3)	0–2.4	0.25		3 (1.2)	0–3.2	0.46
3rd-generation cephalosporin-resistant *E. coli*	2,257 (17.5)	6.1–22.8		284 (17.9)	7.4–19.0	0.66		123 (33.4)	12.7–41.4	<0.01
Quinolone-resistant *E. coli*	4,227 (34.4)	15.2–40.1		453 (28.6)	15.4–40.5	<0.01		150 (41.6)	12.0–54.6	<0.01
Cephalosporin and quinolone-resistant *E. coli*	1,080 (8.4)	0.8–19.9		181 (11.4)	5.7–15.3	<0.01		79 (21.4)	2.9–40.5	<0.01
3rd-generation cephalosporin-resistant *K. pneumoniae*	2,651 (35.9)	9.6–49.7		294 (30.6)	13.8–34.5	<0.01		187 (37.2)	8.8–46.6	0.54
Quinolone-resistant *K. pneumoniae*	3,074 (42.5)	11.5–58.3		321 (33.6)	11.1–39.6	<0.01		183 (36.7)	6.2–47.6	<0.01
Cephalosporin- and quinolone-resistant *K. pneumoniae*	1,839 (24.9)	2.0–46.1		214 (22.3)	6.9–35.2	0.05		135 (26.2)	0.0–41.2	0.47
Carbapenem-resistant *P. aeruginosa*	477 (9.6)	2.4–12.2		45 (16.5)	9.1–23.1	<0.01		74 (18.3)	3.3–27.2	<0.01
Carbapenem-resistant *Acinetobacter* spp.	929 (49.6)	16.9–65.5		86 (48.1)	18.2–66.7	0.66		164 (59.7)	31.6–68.8	<0.01
Multidrug-resistant *Acinetobacter* spp.*	354 (18.2)	3.6–26.1		34 (17.8)	0.0–29.8	0.88		64 (23.4)	0.0–30.2	0.02

*ICU, represents all intensive care units, including surgical, medical, pediatric, and neonatal; *S. aureus, Staphylococcus aureus; E. faecium* or *E. faecalis, Enterococcus faecium or Enterococcus faecalis; E. coli, Escherichia coli; K. pneumoniae, Klebsiella pneumoniae; P. aeruginosa, Pseudomonas aeruginosa*. Multidrug resistant is defined by resistance to ampicillin/sulbactam, carbapenems, all cephalosporins, aminoglycosides (gentamicin and amikacin), and ciprofloxacin. †No. resistant isolates (e.g., methicillin-resistant *S. aureus*, carbapenem-resistant *P. aeruginosa*) from all clinical specimens from all hospitals. The percentage in parenthesis refers to the proportion of resistant isolates over all isolates of the same species (resistant plus susceptible). ‡Range of proportions of resistant isolates over all isolates of the same species obtained from individual hospitals, expressed as percentages. §p value for χ^2^ test comparing proportion of resistant isolates in blood culture and non–blood culture isolates. ¶p value for χ^2^ test comparing proportion of resistant isolates in ICU vs. non–ICU culture isolates.

Despite the relatively small numbers of *Acinetobacter* spp. isolates compared with the other organisms, carbapenem-resistant *Acinetobacter* spp. were found in all ICUs at a high incidence density; as many as 69% of all isolates at 1 ICU were carbapenem resistant. Fully 18.2% of all *Acinetobacter* spp. were resistant to ampicillin/sulbactam, cephalosporins, carbapenems, ciprofloxacin, and aminoglycosides; these particular isolates were susceptible to only the polymyxins. Carbapenem resistance was also found in 9.6% of all *P. aeruginosa* isolates and in up to 27.2% of ICU isolates.

Methicillin resistance occurred in 35.3% of all *S. aureus* isolates. Methicillin-resistant *S. aureus* (MRSA) strains showed correspondingly high resistance levels to macrolides (90.2%), ciprofloxacin (93.9%), and trimethoprim-sulfamethoxazole (49.9%). Vancomycin resistance was reported in 0.8% of all enterococci.

Based on incidence density calculations, MRSA was the predominant drug-resistant pathogen at all hospitals. It had the highest incidence density for blood and ICU cultures (0.31/1,000 inpatient-days and 4.48/1,000 ICU inpatient-days, respectively) among all organisms surveyed. Third-generation cephalosporin-resistant *K. pneumoniae* was the predominant gram-negative resistant pathogen, with an incidence density of 0.19/1,000 and 3.21/1,000 inpatient days for blood and ICU cultures, respectively.

A comparison between organisms isolated from blood cultures and other cultures demonstrated statistically significant differences with regard to percentage resistance for *S. aureus*, *P. aeruginosa*, and the Enterobacteriaceae. The reason for these finding is not evident. In general, ≈10% of all resistant organisms were isolated from blood cultures.

## Conclusions

This is the first comprehensive national survey of antimicrobial drug resistance in Singapore public hospitals. We believe that our findings represent the endemic antimicrobial drug resistance situation in our hospitals; quarterly data analysis did not show any overt outbreak. These results, although new, are not surprising. Previous regional surveys and local studies had already hinted at the extent of the problem in Singapore (*10*–*12*). Similar data have also been reported from other countries in the Asia Pacific region (*10*,*11*).

Use of both incidence density and percentage resistance enabled a more nuanced analysis of the scale of the problem. Although almost half of all *Acinetobacter* spp. clinical isolates were resistant to imipenem, the relative rarity of isolating this organism from clinical specimens renders it a smaller problem compared with MRSA or quinolone-resistant Enterobacteriaceae outside the ICU setting.

In comparison with similar data from Europe (*5*) and Australia (*4*), prevalence of resistance in gram-negative organisms is much higher but prevalence of vancomycin-resistant enterococci is lower. MRSA rates are comparable to those in some countries in southern Europe (*5*) but higher than those in Australia. The reasons for the differences in antimicrobial drug–resistant patterns might be related to infection control practices or to timing of the introduction of resistant organisms. However, more research is needed to clarify these differences.

There are several limitations of this work. First, the inability to segregate nosocomial and community infections prevented a more detailed analysis of antimicrobial drug–resistance issues pertaining to community and hospital settings. Second, the use of different laboratory standards and methods potentially adds a degree of inaccuracy in the analyses. Third, routine laboratory data did not enable us to distinguish the different mechanisms of resistance, particularly among gram-negative bacteria, or to determine the presence of any predominant clone responsible for the high endemic levels of antimicrobial resistance.

Nevertheless, the results can serve to direct any national effort aimed toward reducing the antimicrobial resistance problems of local hospitals. The issues of MRSA in general and carbapenem-resistant *Acinetobacter* spp. and *P. aeruginosa* in local ICUs are particularly pressing. Continued surveillance will also serve as an impartial feedback on the efforts of infection control programs for the future. For a small city-state, comprehensive national surveillance is relatively easier for Singapore than for larger countries. Such surveillance of clinical microbiology isolates is a critical first step toward controlling the growing worldwide threat of antimicrobial drug resistance, and WHONET is a useful tool in this respect.
